# Human CD34^+ ^cells mobilized by granulocyte colony-stimulating factor ameliorate radiation-induced liver damage in mice

**DOI:** 10.1186/scrt22

**Published:** 2010-07-15

**Authors:** Ning Li, Li Zhang, Huixiang Li, Baijun Fang

**Affiliations:** 1 Center of Excellence in Tissue Engineering, Henan Institute of Haematology, Henan Tumor Hospital, Zhengzhou University, 127 Dongming Road, Zhengzhou 450008, China; 2 Department of Medical Oncology, Cancer Center of Sun Yat-Sen University, 651 Dongfeng East Road, Guangzhou 510060, China

## Abstract

**Introduction:**

On the basis of the recently recognized potential of hematopoietic stem cells (HSCs) to give rise to hepatocytes, we have assessed the potential of granulocyte colony-stimulating factor (G-CSF)-mobilized bone marrow-derived CD34^+ ^HSCs to contribute to faster recovery and promote regeneration process after acute liver injury by radiation.

**Methods:**

G-CSF-mobilized CD34^+ ^HSCs (1 × 10^5 ^cells per mouse) were injected via tail vein in the irradiated femal nonobese diabetic/severe combined immunodeficient mice. Irradiated control animals received only saline infusion.

**Results:**

The mobilized CD34^+ ^HSCs significantly ameliorated radiation-induced liver damage. In the liver of recipient mice killed 21 days after irradiation, human albumin^+ ^Y-chromosome^+ ^hepatocyte-like cells, or human cytokeratin^+ ^Y-chromosome^+ ^hepatocyte-like cells formed cords of hepatocytes, occupied ~30% of the 4-μm section surrounding portal tracts. Furthermore, human-specific albumin mRNA expressed in the liver and human albumin was detected in the serum only in the CD34^+ ^HSC-treated mice.

**Conclusions:**

Treatment with G-CSF-mobilized CD34^+ ^HSCs from bone marrow into peripheral blood could significantly promote tissue reparation after acute liver injury by radiation in mice, possibly by the ability of CD34^+ ^HSCs to generate hepatocytes. So mobilization of CD34^+ ^HSCs might offer a novel therapeutic approach for the treatment of radiation-induced complications after radiotherapy or other acute liver diseases in humans.

## Introduction

During radiotherapy, the most important dose-limiting factor is sensitivity of the normal tissue lying in the radiation field. Even with the most optimal radiation schedule, damage still occurs in normal tissues.

Clinically, radiotherapy has played a limited role in the treatment of malignant intrahepatic cancers owing to the low tolerance of liver to radiotherapy. Radiation-induced liver damage had been observed in 5-10% of patients, who had received radiation doses exceeding 30 Gy [1,2]. In addition, liver transplantation is the only current therapeutic modality for liver failure, but owing to the shortage of organ donors, it is available to only a small proportion of patients. Adult stem cell therapy could solve the problem of degenerative disorders, including liver disease, in which organ transplantation is inappropriate or there is a shortage of organ donors. This view is predicated upon the evidence that stem cells, particularly those in hematopoietic tissue, have the ability to develop into endodermal, mesodermal, and ectodermal cell types [3].

Hematopoietic stem cells (HSCs) have been used for hematological reconstitution for many years. Recently, however, homing and engraftment of HSCs in damaged nonhematopoietic organs, such as vascular tissue [4], myocardium [5-9], brain [10,11], liver [12-14], kidney [15], lung [16,17], skin [17], and salivary glands [18] have been observed and were suggested to contribute to the wound-healing process. In some tissues such as myocardium [6,8,9,19] and liver [12], even improved function has been observed. These studies have provided the proof of principle of damage repair by the application of HSCs. A clinically attractive approach is to use granulocyte colony-stimulating factor (G-CSF) to mobilize HSCs to the circulation, and it has been reported that G-CSF administration after partial orthotopic liver transplantation greatly improved survival rate and liver regeneration of partial graft, partly by its mobilizing HSCs into the injured liver to differentiate into hepatocytes through hepatic oval cell engraftment. However, the role of G-CSF-mobilized HSCs in the regeneration of radiation-induced liver injury is unknown and is less understood for this process.

In the current study, we investigated the possibility that G-CSF-mobilized CD34^+ ^HSCs could home to the injured liver and promote tissue repair. We also primarily examined the origin of cells reconstituting liver after acute injury by radiation in mice.

## Materials and methods

### Animals

Six- to 8-week-old female nonobese diabetic/severe combined immunodeficient (NOD/SCID) mice were purchased from the Animal Breeding Center of the Peking University (Beijing, China). All mice were bred and maintained under defined flora conditions in individually ventilated (high-efficiency particle-arresting filtered air) sterile microisolator cages (Techniplast, Milan, Italy). All animal handling and experimental procedures were approved by the Animal Care and Use Committee of Zhengzhou University.

### Isolation of human HSCs

G-CSF-mobilized peripheral blood cells were obtained from leukaphereses processed by using a continuous flow cell separator Fenwal CS 3000 (Baxter, Deerfield, IL, USA). Informed consent and local research ethics committee approval were granted in all cases. After the mononuclear cell fraction was collected, CD34^+ ^cells were isolated using the CD34^+ ^positive cell selection kit (MiniMacs; Miltenyi Biotec, Bergisch Gladbach, Germany).

### Radiation-induced liver damage and G-CSF-mobilized CD34^+ ^HSCs administration

Female NOD/SCID mice were randomly divided into three groups (n = 20 in each group). Animals in Groups 1 and 2 were anesthetized using injection of ketamine (Yuhan Co., Seoul, Korea; 50 mg/kg, i.p.) and xylazine (Bayer, Ansan, Korea; 5 mg/kg, i.p.) prior to immobilization in the recumbent position on a treatment table. The liver portion was centered in a 2 × 4-cm exposure field with the other abdomen, and the body was protected using customized lead shielding. Then the mice were subjected to a single dose of irradiation of 260 cGy with a cesium source (MDS Nordion; Gammacell, Ottawa, ON, Canada). After this, G-CSF-mobilized human CD34^+ ^cells (1 × 10^5 ^cells per mouse) were injected via tail vein in Group 1 (G1) immediately after irradiation. Animals in Group 2 (G2) received the same volume of saline and served as controls. Mice in Group 3 (G3) served as normal controls. After 7 or 21 days to irradiation, mice were killed under anesthesia. The left lobe of the liver was fixed in 4% paraformaldehyde and paraffin-embedded for sectioning. Other portions of liver were frozen in liquid nitrogen and kept at -70°C for preparation of total RNA. Blood was collected and serum separated to evaluate alanine aminotransferases (ALT) and aspartate aminotransferase (AST) activity [20].

### Histological examination

In all experimental groups, 4-μm-thick sections of formalin^-^fixed and paraffin-embedded livers were routinely processed for hematoxylin and eosin staining to determine the extent of liver necrosis/degeneration development. Several fields in each slide were randomly selected, and representative results from 10 animals are shown.

### Immunohistochemistry

Tissue samples from liver were embedded in paraffin wax and cut into 4-μm serial sections. After being deparaffinized with xylene, tissue sections were rehydrated. Endogenous peroxidase activity was quenched with 0.6% hydrogen peroxide for 8 minutes at room temperature. Tissues were then digested with pepsin (Biogenex, San Ramon, CA, USA) for 10 minutes at room temperature and washed in phosphate-buffered saline (PBS). After blocking, slides were incubated for 30 minutes at 37°C with rabbit anti-human cytokeratin monoclonal antibody (BACHEM, Bubendorf, Switzerland) or rabbit anti-human albumin antibody (DAKO, Glostrup, Denmark). Incubation with secondary biotinylated goat anti-rabbit antibody (Vector Laboratories, Burlingame, CA, USA) was performed after being washed, followed by incubation with avidin-biotin complex for 30 minutes at room temperature. Diaminiobenzidene (DAB; Sigma) was used as the visualizing agent. Sections were counterstained with hematoxylin.

### Immunofluorescent staining and fluorescent in situ hybridization (FISH)

The slides of 4-μm frozen liver sections were deparaffinized in xylene (two times) for 10 minutes, rehydrated, blocked with 5% donkey serum for 10 minutes, then incubated for 1 hour with diluted rabbit anti-human ALB antibody (DAKO) or rabbit anti-human cytokeratin antibody (BACHEM) at room temperature. Samples were washed thoroughly with PBS, incubated for 30 minutes at room temperature with donkey anti-rabbit IgG congujated with PE (Jackson ImmunoResearch, West Grove, PA, USA). After being washed with PBS, slides were blocked again with a 5% normal rabbit serum for 10 minutes and then localized with diluted monoclonal Cy5-conjugated anti-human CD45 IgG1 antibody (BD Biosciences Pharmingen, San Diego, CA, USA) for 1 hour at room temperature. After these procedures, Y-FISH analysis was performed as follows. The slides were washed in PBS for 5 minutes, digested at 37°C with 100 μg/ml RNase A for 30 minutes and then with 0.005% pepsin solution (pH 2.0) for 5 minutes. Human Y-chromosome DNA probe labeled with FITC (CEP Y Spectrum Green, Vysis; Downers Grove, IL, USA) was used according to the manufacturer's instructions. After these, the slides were observed field by field using confocal fluorescence microscopy (Confocal 1024 microscope; Olympus AX70, Olympus Optical Co. Ltd.). We screened 40 serial sections of each mouse liver for positive human cells to determine the number of differentiated cells. A minimum of 100 high-power fields in tissues from transplanted animals were analyzed.

### Reverse transcriptase-polymerase chain reaction (RT-PCR) analysis

Total RNA was prepared with TRIzol Reagent (Invitrogen, Carlsbad, CA, USA) according to the manufacturer's protocol (Gibco Life Technologies, Paisley, UK). Normal human liver tissue obtained from normal areas during the surgical resection of hepatocellular carcinoma was used as a positive control. RNA was purified to remove contaminating DNA according to the DNA-free protocol (Ambion, Austin, TX) and quantified using a spectrophotometer. Equal amounts of RNA were used for the synthesis of cDNA. Oligonucleotide primers were human albumin (ALB, 380 bp), forward, 5'-AGC GGC ACA GCA CTT CTC TCT AGA-3', reverse, 5'-TCC ACA CGG AAT GCT GCC ATG G-3'; human cytokeratin 19 (CK19, 328 bp), forward, 5'-ATG GCC GAG CAG AAC CGG AA-3', reverse, 5'-CCA TGA GCC GCT GGT ACT CC-3'; human glutamine synthetase (GS, 397 bp), forward, 5'-GTC AAG ATT GCG GGG ACT AA-3', reverse, 5'-TAC GAT TGG CTA CAC CAC CA-3'; human β-actin (256 bp), forward, 5'-GGG TCG GAA GGA TTC CTA-3', reverse, 5'-GGT CTC AAA CAT GAT CTG GG-3'; mouse glyceraldehyde 3-phosphate dehydrogenase (GAPDH) (287 bp), forward, 5'-TCC TGC ACC ACC AAC TGC TTA G-3', reverse, 5'-CAG ATC CAC AAC GGA TAC ATT G-3'. The PCR procedure consisted of 30 cycles of denaturation for 60 s at 94°C, annealing for 60 s at 58°C (ALB and CK19) or 60°C (GS), and extension for 90 s at 72°C. The samples were subjected to 35 cycles, and the PCR products were analyzed on an ethidium bromide-stained agarose gel.

### Western blotting

Mouse sera were subjected to electrophoresis with 0.1% sodium dodecyl sulfate-7.5% polyacrylamide gel electrophoresis gel. Separated proteins were transferred to a nitrocellulose membrane (Amersham Pharmacia Biotech, Uppsala, Sweden). Human ALB protein was detected by immunoblotting using monoclonal antibody against human ALB (clone 4761; Institute of Immunology, Tokyo, Japan) diluted with 0.05% Triton X-100 in Tris-buffered saline containing 1% gelatin, followed by incubation with a goat anti-rabbit horseradish peroxidase-conjugated antibody (1:10,000 dilution) (E.I. du Pont de Nemours, Boston, MA, USA). All membranes were immunoblotted for the mouse GAPDH antibody (Santa Cruz Biotechnologies Inc., Camarillo, CA, USA) as the internal standard protein. Immunodetection using the enhanced chemiluminescence method (ECL kit; Amersham, Piscataway, NJ, USA) was performed according to the manufacturer's instructions. Normal human and nontransplanted murine sera were used as controls.

### Statistical analysis

To assess the statistical significance of intergroup differences in quantitative data, the Mann-Whitney *U *test was used to compare mean values between the two groups. *P *< 0.01 was considered statistically significant.

## Results

### G-CSF-mobilized CD34^+ ^HSCs ameliorate radiation-induced liver injury

**Figure 1:**
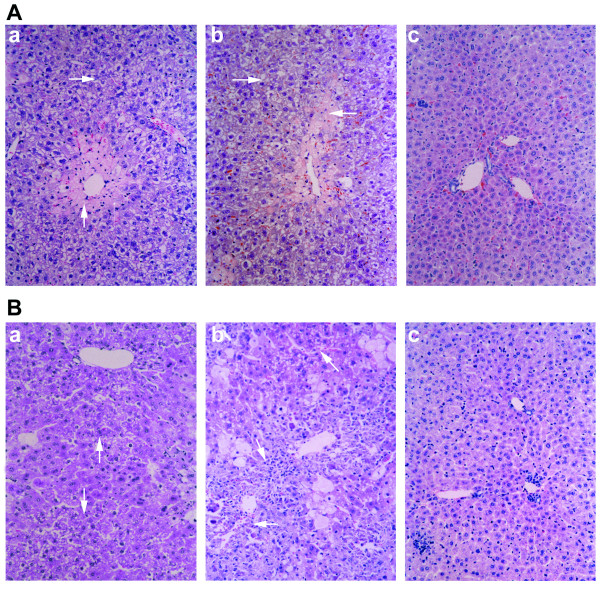
**Improved morphology after liver irradiation with or without transplantation of mobilized CD34^+ ^hematopoietic stem cells (HSCs)**. Representative photomicrographs of the liver at 7 or 21 days after irradiation in **(a) **Group 1 (G1), **(b) **Group 2 (G2), and **(c) **Group 3 (G3) mice. **(d) **Seven days after irradiation, the extent of liver degeneration/necrosis (arrow) was not significantly different between **(e) **G1 and **(f) **G2 mice. Twenty-one days after irradiation, however, areas of liver degeneration/necrosis were smaller in **(g) **G1 mice compared with those in **(h) **G2 mice. Paraffin-embedded sections were processed for hematoxylin and eosin staining. Original magnification, ×20.

The initial challenge to study G-CSF-mobilized CD34^+ ^cells to hepatocyte differentiation was to establish a model of acute liver damage in immunodeficient mice, in which the control mice that did not undergo transplantation would recover from the injury. Hepatic injury in NOD/SCID mice by several different doses of radiation was studied first. All mice could tolerate up to 260 cGy. On day 7 after irradiation, the extent of liver degeneration/necrosis was obvious in all experimental mice, but without significant differences among G1 and G2 mice (Figure 1d). On day 21, however, areas of liver degeneration/necrosis were smaller in G1 mice than in G2 mice (Figures 1g and 1h). In addition, to assess the liver damage induced by irradiation, the level of ALT and AST activity was measured. In G1 and G2 mice, the activity of serum ALT and AST showed initially significantly elevated and gradually decreased with time, and in the G1 mice, both ALT and AST presented significantly lower values when compared with the values found in G2 mice at the same time points (Table 1).

**Table 1:** Effect of G-CSF-mobilized CD34^+ ^cells on murine liver function after irradiation

Mice	ALT (IU/L)				AST (IU/L)				ALB^+^/Y-chromosome^+ ^cells	
			
	0 d	3 d	7 d	21 d	0 d	3 d	7 d	21 d	14 d	21 d
G1	37.9 ± 7.2	418.3 ± 49.8^†^	228.3 ± 32.7*	78.3 ± 5.7*	31.1 ± 6.9	398.3 ± 42.8^†^	256.3 ± 36.8*	89.2 ± 6.4*	_	+^#^
G2	36.4 ± 6.7	434.5 ± 53.8	378.6 ± 51.2	169.3 ± 34.7	29.8 ± 6.3	402.5 ± 46.5	381.2 ± 50.9	209.5 ± 38.4	_	_
G3	38.4 ± 7.1	40.9 ± 6.1	38.3 ± 6.8	42.3 ± 6.7	30.7 ± 6.5	31.7 ± 6.4	30.8 ± 6.9	31.1 ± 6.5	_	_

Data are means ± SD (n = 10). *P < 0.05 compared with the G2 mice; †*P *> 0.05 compared with the G2 mice. #Human ALB^+^/Y chromosome^+ ^hepatocyte-like cells in the liver of G1 mice. G-CSF, granulocyte colony-stimulating factor; ALB, albumin; ALT, alanine aminotransferase aspartate; AST, aspartate aminotransferase.

### G-CSF-mobilized CD34^+ ^HSCs engraft into irradiated mice and differentiate

**Figure 2:**
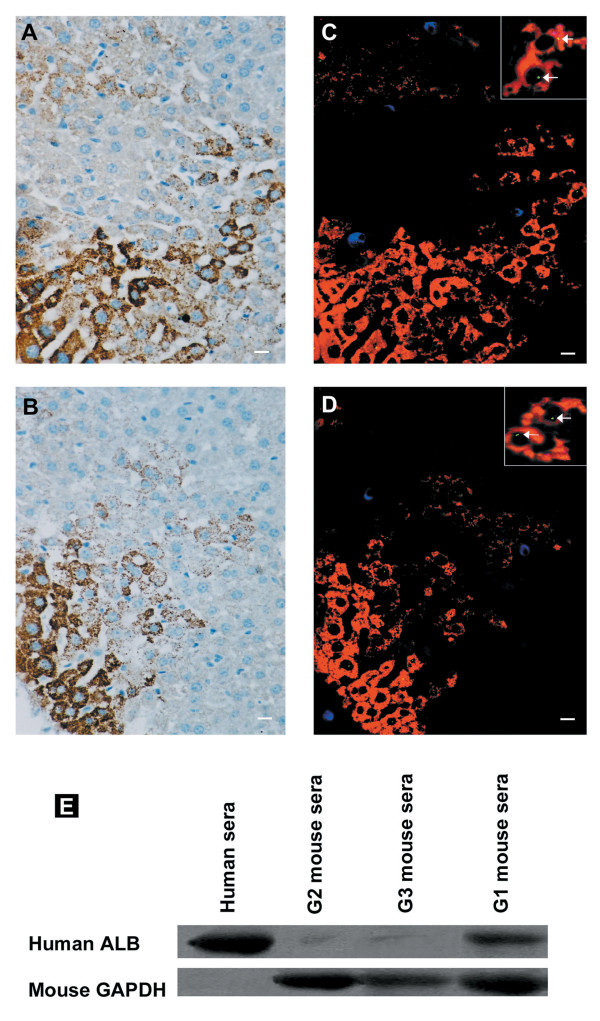
**Engraftment and in vivo differentiation of granulocyte colony-stimulating factor (G-CSF)-mobilized CD34^+ ^HSCs into hepatocyte-like cells in G1 mice 21 days after irradiation and transplantation**. Deparaffinized section of liver was stained with **(a)** anti-human albumin (ALB) antibody or **(b)** anti-human cytokeratin antibody. **(c)** Liver was stained with anti-human ALB antibody (red), anti-human CD45 antibody (blue), and human Y chromosome DNA probe (green). **(d) **Liver was stained with anti-human cytokeratin antibody (red), anti-human CD45 antibody (blue), and human Y chromosome DNA probe (green). The arrow indicates Y chromosome^+^cytokeratin^+ ^or Y chromosome^+^ALB^+ ^cells in the liver of G1 mice. The absence of chromosomal staining in some ALB^+^/cytokeratin^+ ^cell nuclei may be a result of partial sampling of nuclei in the 4-μm thin tissue section. **(e) **Human serum ALB in the plasma of G1 mice was identified by Western blot analysis. Mouse glyceraldehyde 3-phosphate dehydrogenase (GAPDH) acts as internal standard. Original magnification, **(a-d)**, ×20; inset in **(c) **and **(d)**, ×60. Representative images are shown.

Considering the possible continued existence of donor-derived hematopoietic cells in recipient mice, we used triple-color immunofluorescence to discriminate between donor-derived epithelial and donor-derived haematopoietic cells in the same tissue sections. In the liver of G1 mice killed 21 days after irradiation, human ALB (Figure 2A), CK19 (Figure 2B) positive hepatocytes, both human ALB and human Y-chromosome positive hepatocytes (Figure 2C), or both human CK19 and human Y-chromosome positive hepatocyte-like cells (Figure 2D) formed cords of hepatocytes occupying ~30% of a given 4-μm section surrounding portal tracts in a pattern similar to that seen in hepatic regeneration from hepatic oval cells [21,22], but this phenomenon was not found in Groups 2 and 3 mice (data not shown). These results indicate that G-CSF-mobilized CD34^+ ^HSCs might be capable of differentiating into ALB-producing cells in the liver of irradiated recipients.

To evaluate the function of these repopulating cells, we analyzed the production of ALB in donor-derived parenchymal cells. As shown in Figure 2E, we detected human ALB protein in the sera of the G1 mice on day 21 after irradiation. The immunoreactive band of control human serum diluted 1:100 was 103 times stronger than the faint band of nondiluted serum from the nontransplanted control mice (G2 and G3 mice). Since the concentration of ALB in the nontransplanted control mice serum of the same band scarcely changes, the strong immunoreactive bands of sera from G1 mice indicate the presence of human ALB. These observations proved that transplanted G-CSF-mobilized CD34^+ ^HSCs developed into functional hepatocytes.

**Figure 3:**
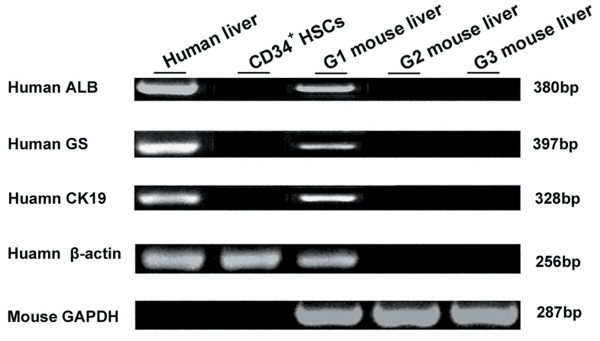
**The expression of human-specific genes of ALB, CK19, and GS by reverse transcriptase-polymerase chain reaction in the mouse liver at 21 days after irradiation**. Representative images are shown.

Mice were killed 7 or 21 days after transplantation with G-CSF-mobilized CD34^+ ^HSCs. RNA samples were obtained from the livers of the mice in each group. RT-PCR was performed to detect human-specific expression of ALB, GS, and CK-19. To demonstrate the specificity of the primers to detect human ALB, GS, and CK19 mRNA, we examined the mRNA in nontransplanted mice livers (G2) and in normal control mice livers (G3). These samples were negative in every assay, and an example is shown in Figure 3. Because of the phenomenon known as "stem cell priming," in which primitive hematopoietic cells have been shown to express low levels of various mRNA before committing to a particular blood cell lineage [23,24], we also tested the G-CSF-mobilized CD34^+ ^HSCs for ALB, GS, and CK19 expression. As shown in Figure 3, the starting CD34^+ ^HSCs did not express ALB, GS, or CK19 when they were freshly isolated. However, in the mice (G1) with radiation-mediated liver injury that received CD34^+ ^HSCs transplantation 21 days later, RT-PCR analysis showed that human GS, CK19 and ALB mRNA were expressed (Figure 3), which meant a portion of the transplanted human stem cells had localized to the liver and had begun to express human ALB when analyzed 21 days after the liver damage.

## Discussion

The plasticity studies reported so far in the literature have demonstrated that HSCs can differentiate to tissues other than blood [4-19]. In recent clinical practice, circulating HSCs numbers can be increased by mobilization from the bone marrow with G-CSF [25,26], and the mobilization of HSCs by G-CSF has been shown to be involved in the reparation of the infarcted heart [8,27]. Therefore, we decided to address whether G-CSF-mobilized HSCs could contribute to faster recovery and promote the regeneration process after acute liver injury by radiation. The present study provides the first evidence that mobilization of G-CSF-mobilized CD34^+ ^HSCs after liver irradiation has beneficial effects in preventing and/or reducing radiation-induced damage. In our acute radiation-induced liver injury model, G-CSF-mobilized CD34^+ ^HSC administration ameliorated the histological damage and accelerated the regeneration process. In all animals sacrificed 21 days posttransplantation, immunohistochemical analysis showed human ALB/cytokeratin-positive cells in mobilized CD34^+ ^HSC-treated mouse liver. FISH analysis using the human Y chromosome also showed positive signals. In contrast, immunopositive cells were not detected in the irradiated control group. Furthermore, human-specific ALB mRNA was expressed in the liver, and human ALB was detected in the serum only in the mobilized CD34^+ ^HSCs-treated mice. These observations prove that transplanted G-CSF-mobilized CD34^+ ^HSCs might develop into functional hepatocytes.

Recently, cell fusion was suggested as an explanation for stem cell plasticity. Some scholars raise doubts about whether transdifferentiation or dedifferentiation actually occurs [28-30]. Their findings in vitro or in vivo suggested that stem cell "transdifferentiation" or "plasticity" is a result of cell fusion events. In this study, we did not determine whether fusion of G-CSF-mobilized CD34^+ ^HSCs with liver cells was responsible for their hepatocyte-like morphology or whether the cells change their fate in vivo in response to local cues after transplantation.

So the question remains as to what is the role of G-CSF-mobilized HSCs in the regeneration of radiation-induced liver injury and what is the regenerative process. Recently, Yannaki *et al*. [31] indicated that treatment with G-CSF-mobilized HSCs significantly improved survival and liver histology in chemically injured mice, predominantly by promoting endogenous repair mechanisms. Similar regenerative processes have been reported in organisms such as salamanders and *Caenorhabditis elegans*. In addition, bone marrow-derived stem cells could initiate pancreatic regeneration [32]. However, in our study, the percentage of G-CSF-mobilized CD34^+ ^HSC-derived hepatocyte-like cells present in sections of irradiated hepatic tissues was much higher than that reported by Yannaki *et al*. [31]. So we suggest it might be G-CSF-mobilized CD34^+ ^HSCs that ameliorate radiation-induced damage to liver by the ability to generate hepatocytes, although we still could not exclude the possibility that endogenous hepatic stem cells are also involved in regenerative processes at the same time. However, despite the obscurity of the cellular mechanism, the ability of G-CSF-mobilized HSCs to reestablish physiological function in the whole animal has been documented [6,8,18,19,31].

## Conclusions

In summary, our data suggest that G-CSF-mobilized CD34^+ ^HSCs, given their potential to engraft into liver tissue and their ability to ameliorate the injurious effects of radiation, might provide a new approach for the treatment of some liver diseases. This study indicates a clinical applicable protocol for the use of HSC mobilization to ameliorate radiation-induced damage.

## Abbreviations

ALB: albumin; ALT: alanine aminotransferase aspartate; FISH: fluorescence in situ hybridization; G-CSF: granulocyte colony-stimulating factor; HSCs: hematopoietic stem cells; NOD/SCID: non-obese diabetic/severe combined immunodeficient.

## Competing interests

The authors declare that they have no competing interests.

## Authors' contributions

NL, LZ, and BF contributed to the conception and design, interpretation of data, and drafted the manuscript. NL, LZ, and HL performed research and collected data. All authors read and approved the final manuscript.
